# Machine-learning for quantitative histopathology of piglet intestinal tissues: challenges with limited training data

**DOI:** 10.3389/fvets.2025.1620338

**Published:** 2025-10-06

**Authors:** Cecilie Brandt Becker, Mette Sif Hansen, Søren Saxmose Nielsen, Henrik Elvang Jensen

**Affiliations:** ^1^Section for Parasitology and Pathobiology, Department of Veterinary and Animal Sciences, Faculty of Health and Medical Sciences, University of Copenhagen, Copenhagen, Denmark; ^2^Section for Animal Health and Welfare, Department of Veterinary and Animal Sciences, Faculty of Health and Medical Sciences, University of Copenhagen, Copenhagen, Denmark

**Keywords:** digital pathology, intestinal segmentation, machine learning, histopathology, pigs

## Abstract

**Introduction:**

Use of Machine learning (ML) is rapidly expanding in histopathology, offering the potential to reduce interobserver variability and improve quantitative assessment. However, large datasets and computational resources commonly used in toxicology and human medicine are often unavailable to the veterinary pathologist. This study aimed to evaluate the feasibility and limitations of applying supervised ML on histopathological samples with limited training data, exemplified by training an ML model to segment the intestinal wall into its histological layers.

**Materials and methods:**

The study included 145 piglets from five age groups (4, 14, 25, 49, and 67 days). Full-wall samples from duodenum, jejunum and ileum were collected post-mortem, stained with H&E and digitized. A three-step ML model was trained on 8–15 images: Step 1 identified tissue, Step 2 segmented mucosa from submucosal layers, and Step 3 separated lamina propria from epithelium. Model performance was assessed by comparing AI-generated areas to manual annotations, calculating relative deviation, categorized agreement levels, Intersection over Union, and Pearson correlation coefficients. Qualitative error analyses were used as directions for future training options.

**Results:**

A three-step separation model was successfully developed, but showed a significant amount of age-related performance variation, depicted as larger inaccuracies in samples from the younger age-groups, reflecting additional tissue heterogeneity from immature morphology. Classification errors could be categorized into intrinsic limitations (e.g., thresholding issues in tissue identification) and training deficits (e.g., misclassification of goblet cells and crypt abscesses), of which only the latter category could be corrected by adding additional training data.

**Conclusion:**

This study demonstrates the feasibility of ML-based histopathology with limited sample sizes, providing a viable option for veterinary pathologists. Models trained on small datasets require careful supervision, with special emphasis on age-diverse tissue heterogeneity and overfitting. In these cases, ML should be seen as a tool to augment, not replace, expert oversight, ensuring reliable and reproducible quantitative histopathological measures.

## 1 Introduction

Porcine intestinal disease, and in particular post-weaning diarrhea (PWD), is a significant animal welfare issue (1, 2), which presents challenges due to the extensive use of antibiotics to treat affected animals, which contributes to the development and spread of antimicrobial resistance (3, 4). The causes of PWD are complex and multifactorial (5), necessitating thorough investigations to elucidate the pathological mechanisms underlying diarrhea development, and to aid determining when antibiotic treatment might be beneficial. However, histopathological lesions are often inconsistent in PWD pigs and may lack a clear correlation with clinical signs (6). Additionally, previous studies have faced limitations due to rapid autolytic changes in the intestinal mucosa post-mortem, which have hindered the interpretation of villus-associated lesions (6–8). Moreover, histopathological assessment of intestinal pathology has traditionally relied on descriptive and semiquantitative observations prone to interobserver variability, even among field specialists (9–11). To minimize this variability, observations have often been grouped into dichotomous or semiquantitative categories (6, 12); however, this approach potentially oversimplifies the complexity of PWD and may obscure subtle differences that could be crucial for understanding the underlying disease mechanisms. In recent decades, the increased application of digital pathology, coupled with rapid advancements in artificial intelligence (AI) and machine-learning (ML), has transformed histopathological workflows in human diagnostics, research, and toxicology, shifting towards more quantitative and objective measurements enabled by computer-assisted technologies (13–15). Although these technologies have made their way into human gastroenterology among others (13, 16, 17), the application of computational histology, remains in its infancy within the field of veterinary medicine (18). This slower progress in veterinary applications is probably in part due to the substantial sample sizes normally required to train reliable classifiers, which poses a significant challenge (18, 19). Unlike in human medicine, where larger datasets are often readily available (20), veterinary research frequently faces limitations in both sample size and data heterogeneity, making it difficult to develop robust, generalizable models for histopathological assessment. Additionally, applying machine-learning techniques often requires significant computational power, which can pose yet another challenge for many veterinary research settings (18).

The aim of this study was to evaluate whether a supervised machine-learning model could be developed to accurately separate the histological layers of the piglet intestinal wall using the small and heterogeneous datasets common in veterinary research. The model was created as a prerequisite for future studies of post-weaning diarrhea (PWD). Our objectives were to determine the extent to which accurate segmentation is achievable under these constraints, and to highlight key limitations that impact model performance. By addressing these points, this study provides practical insights for applying machine learning in histopathology when large datasets and high-performance computing are not available.

## 2 Materials and methods

### 2.1 Animals

This study utilized tissue samples from 145 piglets reared in a conventional indoor intensive production herd in Denmark. Included animals were not subjected to litter equalization or cross-fostering, and all piglets were weaned at 26 days of age. The animals were randomly selected from a cohort of 2,500 piglets representing individuals both with and without signs of gastrointestinal disease. The PWD disease prevalence in the study was 43%. The piglets were stratified into five different age groups: 4, 14, 25, 49, and 67 days.

### 2.2 Sample collection

Between July 2023 and March 2024, samples were collected in 11 batches, each containing 10–15 animals. On the morning of sampling, all piglets were transported for approximately 1.5 h from the farm to Frederiksberg Campus, University of Copenhagen. The piglets were anesthetized via intramuscular injection of Zoletil (0.1 mL/kg) and euthanized by intracardiac injection of an overdose of pentobarbital. Following evisceration, full-thickness samples of mid-jejunum were collected from all animals, while samples from duodenum and ileum were taken from a subset of 75 animals representing all age groups. Duodenum was identified as the segment between the pylorus of the stomach and the oral aspect of the duodenocolic fold. Jejunal samples were taken from mid-jejunum, approximately midway between the aboral edge of the duodenocolic fold and the ileocecal fold. Ileal samples were identified as the segment between the oral edge of the ileocecal fold and the ileal inlet to cecum. The samples were opened, gently rinsed in isotonic saline, and pinned onto styrofoam boards before being immersed in 10% neutral buffered formalin. All samples were secured in fixative within 15 min of euthanasia.

### 2.3 Tissue processing

Samples were immersion-fixed in 10% neutral buffered formalin for 3 days. After fixation, tissues were trimmed and processed through graded concentrations of ethanol and xylene, embedded in paraffin, and samples of 5 μm were stained with hematoxylin and eosin (H&E) using standard protocols as previously described (6).

### 2.4 Digitization of slides

Stained histological slides were digitized for image analysis by whole-slide scanning with a Zeiss Axioscan Z1 scanner, using a 20x/0.8 Plan-Apochromat objective. Software settings were adjusted specifically for brightfield microscopy of 5 μm H&E stained intestinal tissues.

### 2.5 Model development

**Figure 1:**
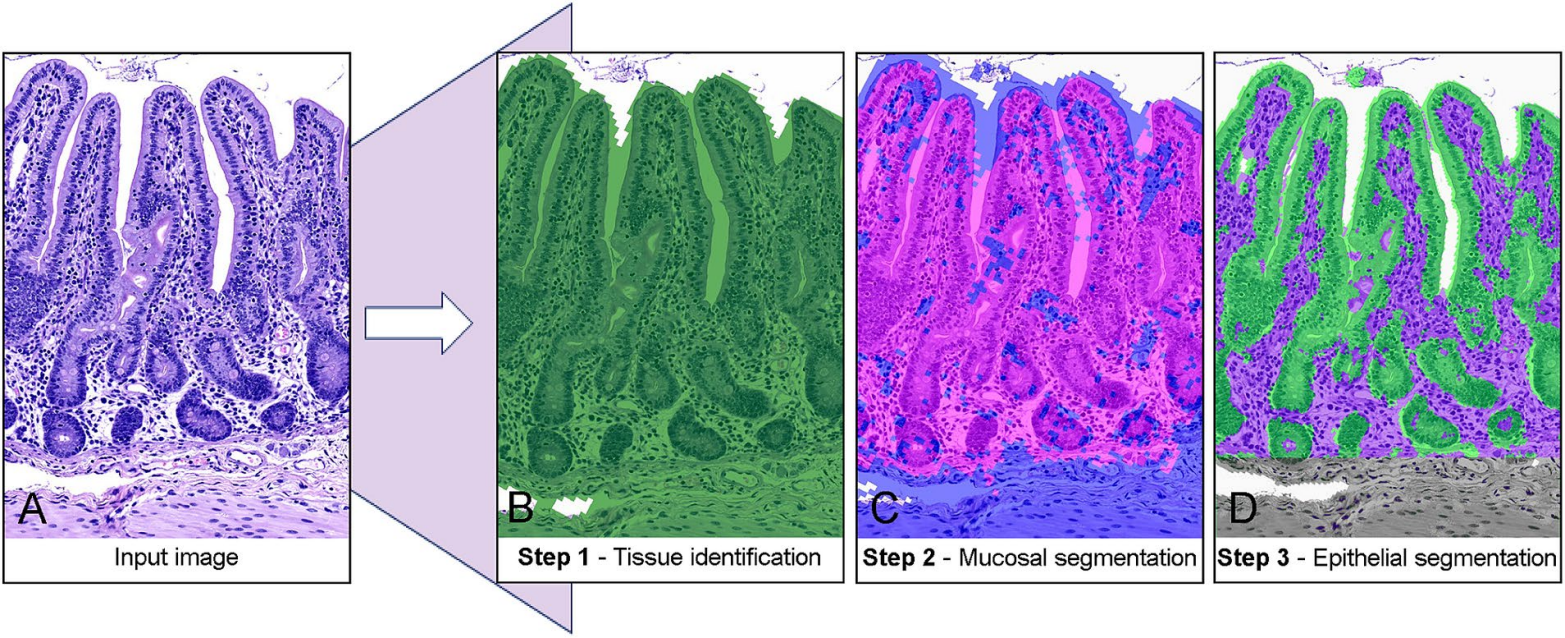
Stepwise classification of porcine jejunal tissue using machine-learning-based segmentation. **(A)** Raw input image of an H&E-stained tissue section. **(B)** Step 1: Tissue identification with a pixel thresholder separates tissue (green) from the background. **(C)** Step 2: Mucosal segmentation. A random-trees pixel classifier distinguishes the mucosa (pink) from the submucosa and muscular layers (blue). **(D)** Step 3: Epithelial segmentation. A random-trees pixel classifier isolates the epithelium (green) from the lamina propria (purple).

Image pre-processing and ML model training were conducted on a Lenovo ThinkPad laptop with an Intel Core i5 processor, to mimic the commonly available hardware setup in veterinary research settings. Model-training and image analysis was conducted using the open-source image analysis software QuPath [version 0.5.1 (21),]. Model training was performed exclusively using jejunum samples to ensure consistency in tissue structure and morphology. Prior to analysis, all tissue sections were individually color-normalized to H&E using the built-in “Estimate Stain Vectors” tool. To achieve complete segmentation of the intestinal wall layers, a 3-step separation model was developed (Figure 1). All settings adjusted for model development are mentioned in the following paragraphs. If not mentioned, the settings were left as default.

In the first step, tissue was automatically identified and annotated using a pixel-based thresholding approach. This was achieved by analyzing the average RGB values (i.e., Red-Green-Blue values) of each pixel at a resolution of 7.03 μm/px, with a Gaussian prefilter and a smoothing sigma of 2.0 applied. A threshold value of 205 was used as cutoff, with pixels below this value classified as tissue and those above classified as background (Figure 1B). In order to measure the tissue area, the classification was converted to an object using the “create objects” function, with minimum object size of 10,000 μm^2^ and a minimum hole size of 100,000 μm^2^ to avoid misclassification of vascular structures and edematous areas.

In the second step, a machine learning-based pixel classifier, utilizing the default random trees algorithm, was trained to differentiate the intestinal mucosa from the underlying submucosa and muscular layers (Figure 1C). Classification was performed based on the RGB and Hematoxylin channels of the image, with a resolution of 7.03 μm/px. The classifier employed features of Gaussian, Laplacian of Gaussian, Weighted Deviation, and Hessian Determinant, with scaling factors of 1.0 and 2.0, a selection based on published guidelines (22). Training images were blinded with regards to disease status and added *ad hoc* by continuous inclusion of problematic slides, and the final model was developed using 236 manual annotations of “mucosa” and “submucosa/muscularis” from 15 distinct animals (n_4_ = 2; n_14_ = 2; n_25_ = 2; n_49_ = 8; n_67_ = 1). All annotations were made by the main author (CBB) using the built-in brush-tool in QuPath to provide the software with examples of mucosal and submucosal tissues, respectively. Area extraction was achieved by applying the same approach as described for Step 1.

In the final step, another pixel classifier utilizing a random trees algorithm was trained to differentiate the epithelium from the lamina propria within the mucosal area identified previously by the model (Figure 1D). As for the second step, classification was based on the RGB and Hematoxylin channels, but at a higher resolution of 3.51 μm/px. To address the increased complexity of the structures, the classifier incorporated additional features including Gaussian, Laplacian of Gaussian, Weighted Deviation, Gradient Magnitude, Structure Tensor Coherence, and Hessian Determinant, all with a scaling factor of 1.0. The training set comprised eight distinct tissue samples included *ad hoc*, and 172 manual annotations (n_4_ = 0; n_14_ = 1; n_25_ = 2; n_49_ = 3; n_67_ = 2) made in a similar fashion as for Step 2. For area extraction a minimum object size and minimal hole size of 1,000 μm^2^ was applied.

To account for potential age-related differences in tissue morphology, Steps 2 and 3 were repeated, and separate classifiers were trained on new images using age-balanced datasets with all age-groups represented in the training data. The age-balanced training for Step 2 again included 15 tissue samples and 236 annotations (n_4_ = 2; n_14_ = 2; n_25_ = 2; n_49_ = 8; n_67_ = 1), while Step 3 utilized 10 tissue samples and 285 annotations (n_4_ = 2; n_14_ = 2; n_25_ = 2; n_49_ = 3; n_67_ = 2). The classifier specifications, including feature sets and scaling factors, remained unchanged.

### 2.6 Performance evaluation and statistics

The performance of the trained models, including both the *Ad Hoc* trained and the Age-balanced models, was assessed by comparing the areas predicted by each step of the classification process (*A_model_*) to manually annotated areas (*A_manual_*, applied as ground truth), across all 145 jejunal samples. In general terms, the above mentioned samples (15, 8, and 10 samples respectively) were selected as training sets, while the whole dataset of 145 animals were used for validation. No separate dataset for a final testing phase was available in this study. For each sample, a 5 mm section containing longitudinally sectioned crypts of Lieberkühn was selected for validation. The length of the section was determined by measuring along the lamina muscularis mucosa using the polyline tool. The relative deviation from the manual annotations was calculated as follows:


$$\text{Relative deviation}\;(\mathrm{RD})=\frac{A_{\text{model}}-A_{\text{manual}}}{A_{\text{manual}}}\times 100\%$$

Differences in relative deviation across age groups were visualized using a modified Bland–Altman plot and categorized according to level of agreement. Agreement levels were defined as follows: RD < 5% = Very good, RD = 5–10% = Good, RD = 10–20% = Fair, and RD > 20% = Poor. The performance of each classification step was further evaluated within the five age groups by calculating the Pearson correlation coefficient between manually annotated areas and areas provided by the trained models (23).

To assess spatial agreement between manually annotated regions and AI-generated segmentations, Intersection over Union (IoU) was calculated. This metric quantifies the proportion of overlap between the two areas relative to their combined area, thereby verifying that segmented regions were co-located rather than merely adjacent. IoU values were interpreted using commonly applied thresholds, with values >0.50 considered acceptable, >0.75 considered good, and >0.90 considered near-perfect. The QuPath script used for IoU calculation is provided in the Supplementary material. In addition to the quantitative evaluation, a visual inspection of classification errors was performed to identify recurring misclassifications and limitations in the applied methodology. Tissue structures that were consistently misclassified in the different steps of the classification process were recorded in order to guide potential adjustments in future training iterations.

To assess the translatability and adaptability of the *Ad Hoc* trained model to other intestinal regions than the jejunum assessed above, three samples from both duodenum and ileum from each of the five age groups were evaluated. A section measuring 2.5 mm of each sample was evaluated as described above. The selection of segments for analysis followed the same principles as for jejunal samples, but actively avoiding segments containing Peyer’s patches in ileum. The model’s performance on these samples was compared to its performance on the jejunum samples from the same piglets, providing insight into its ability to generalize across different anatomical regions in the small intestine.

All statistical analyses were performed using R Statistical Software [R version 4.4.0 (2024-04-24)] (24). Prior to data analysis all parameters were evaluated for normality by visual inspection of histograms.

### 2.7 Ethics declarations

The study was approved by the Danish Animal Inspectorate (License no: 2022-15-0201-01324).

## 3 Results

All parameters were deemed to follow a normal distribution and were handled accordingly in the following sections.

### 3.1 Step 1—tissue detection

**Figure 2:**
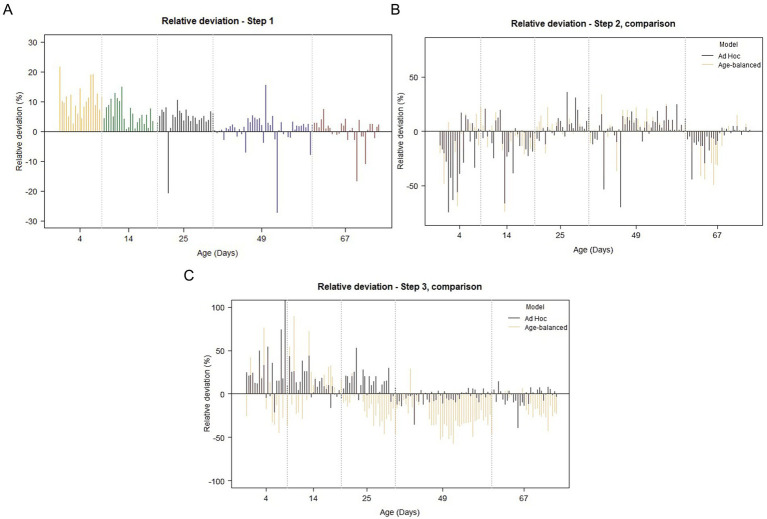
Fluctuations in relative deviation across age-groups for the 3-step separation model (modified Bland–Altman plots). **(A)** Classification step 1. Apart from a few outlier values, note the gradual reduction in relative deviation with increasing age. **(B)** Classification step 2. Larger values of relative deviation are seen in step 2 when compared to step 1, and a similar pattern of variation is seen when comparing the *Ad Hoc*-trained model (black) to the Age-balanced model (orange). In step 2, the effect of increasing age on the magnitude of the relative deviation is less obvious. **(C)** Classification step 3. As for step 1 and 2, the largest relative deviations are seen in the youngest age groups (4–14 days), and a clear decrease in the variation is seen with increasing age for the *Ad-Hoc* trained model. A similar pattern cannot be recognized for the Age-balanced model.

**Figure 3:**
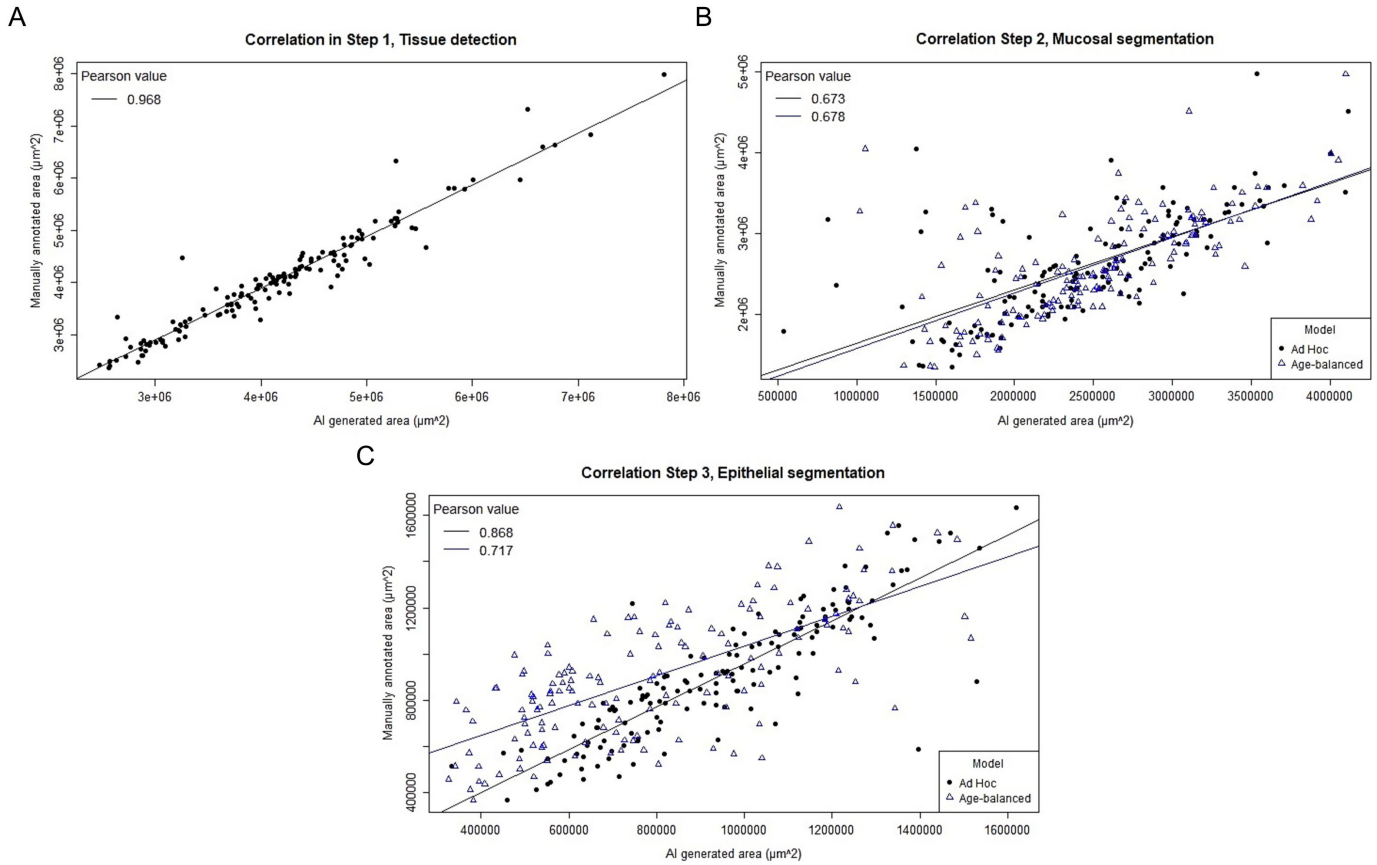
Scatterplot of the correlation between AI-generated and manually annotated areas for the three steps in the separation model. **(A)** Step 1. A very strong positive correlation is seen between manual and AI-generated areas with a Pearson correlation coefficient of 0.968. **(B)** Step 2. A strong, but slightly less convincing correlation is seen between AI-generated and manual areas for step 2. The *Ad hoc* trained model (black dots) and the Age-balanced model (blue triangles) yield similar results. **(C)** Step 3. The correlation between manual and AI-generated areas is very strong for the Ad hoc trained model (black dots), while slightly less pronounced for the Age-balanced model (blue triangles).

Step 1 (tissue detection) demonstrated high accuracy and consistency across all age groups. Mean relative deviations ranged from 2.9 to 10.7%, with the highest deviations observed in the youngest age group (4 days), and gradually increasing performance with increasing age (Figure 2). The Pearson correlation coefficient for tissue detection was very high (0.97), indicating a highly correlated relationship between AI-generated and manually annotated areas across all age groups (Table 1; Figure 3A). When categorized by agreement levels, Step 1 performed robustly, achieving 80% “*very good*” agreement in the oldest group (67 days) and 73% in 49-day-old piglets. However, in the youngest age group (4 days), only 30% of cases achieved “*very good*” agreement, and 30% were classified as either “*fair*” or “*poor*” (Table 2). These results show that tissue detection was consistent overall, but with a reduced accuracy for the younger age groups. When assessing the IoU values, the areas provided by the model showed consistently high colocalization with manual annotations (overall *μ* = 0.91, sd = 0.09). Mean IoU values were above 0.90 for all age groups, except the 4-day olds, thus exceeding the threshold generally interpreted as “near-perfect” alignment (Table 1).

**Table 1:** Performance evaluation of the classification steps of each ML-model based on the relative deviation, Interception over Union calculations (IoU), and Pearson correlation coefficient (PCC) between AI-generated areas and manual annotations.

Classification step	Age group (days)	Relative deviation		Pearson correlation		Intersection over union	
		μ	sd	PCC	Interpretation	μ	sd
Step 1	All	5.1	4.8	1.0	Very strong	0.91	0.09
	4	10.7	5.0	1.0	Very strong	0.85	0.06
	14	5.7	4.1	1.0	Very strong	0.91	0.07
	25	5.9	3.6	0.9	Very strong	0.92	0.10
	49	3.2	4.5	0.9	Very strong	0.94	0.10
	67	2.9	3.4	1.0	Very strong	0.93	0.09
Step 2—*Ad Hoc* model	All	12.0	14.3	0.7	Strong	0.79	0.19
	4	24.6	20.9	0.5	Moderate	0.51	0.15
	14	14.1	14.5	0.6	Strong	0.78	0.19
	25	8.7	9.2	0.9	Very Strong	0.84	0.09
	49	10.0	12.8	0.7	Strong	0.84	0.13
	67	7.6	9.3	0.8	Very Strong	0.90	0.16
Step 3—*Ad Hoc* model	All	12.6	16.1	0.9	Very Strong	0.92	0.11
	4	30.1	30.8	0.5	Moderate	0.84	0.11
	14	15.3	12.8	0.9	Very Strong	0.89	0.09
	25	15.1	11.4	0.9	Very Strong	0.95	0.11
	49	5.6	5.7	1.0	Very Strong	0.94	0.10
	67	7.2	7.2	0.8	Strong	0.95	0.11
Step 2—Age-balanced model	All	11.6	12.4	0.7	Strong	0.81	0.15
	4	18.7	15.7	0.6	Moderate	0.67	0.13
	14	13.9	13.8	0.5	Moderate	0.74	0.16
	25	7.4	6.1	0.9	Very Strong	0.85	0.08
	49	9.5	9.7	0.7	Strong	0.86	0.14
	67	11.3	14.5	0.6	Moderate	0.87	0.15
Step 3—Age-balanced model	All	22.9	16.6	0.7	Strong	0.79	0.11
	4	31.2	17.6	0.3	Weak	0.73	0.13
	14	21.4	21.7	0.9	Very Strong	0.78	0.07
	25	19.8	13.1	0.7	Strong	0.76	0.10
	49	28.2	14.3	0.8	Strong	0.80	0.10
	67	13.3	10.9	0.7	Strong	0.84	0.11

μ, mean; sd, standard deviation; PCC, Pearson Correlation Coefficient.

**Table 2:** Summarized counts of categorized model performance levels (relative deviation), subdivided by age group and classification step.

Age group	Classification step	**Agreement level**			
		**Very good**	**Good**	**Fair**	**Poor**
		(RD < 5%)	(RD = 5–10%)	(RD = 10–20%)	(RD > 20%)
4 Days (*n* = 20)	Step 1	6	8	5	1
	Step 2—*Ad Hoc* model	2	4	6	8
	Step 2—Age-balanced model	2	5	8	5
	Step 3—*Ad Hoc* model	2	1	6	11
	Step 3—Age-balanced model	0	2	5	13
14 Days (*n* = 25)	Step 1	21	4	0	0
	Step 2—*Ad Hoc* model	8	5	7	5
	Step 2—Age-balanced model	6	4	12	3
	Step 3—*Ad Hoc* model	7	5	6	7
	Step 3—Age-balanced model	5	6	4	10
25 Days (*n* = 25)	Step 1	16	6	2	1
	Step 2—*Ad Hoc* model	13	6	3	3
	Step 2—Age-balanced model	13	6	5	1
	Step 3—*Ad Hoc* model	4	7	9	5
	Step 3—Age-balanced model	3	4	7	11
49 Days (*n* = 45)	Step 1	33	5	6	1
	Step 2—*Ad Hoc* model	18	15	8	4
	Step 2—Age-balanced model	18	14	7	6
	Step 3—*Ad Hoc* model	26	14	4	1
	Step 3—Age-balanced model	3	3	7	32
67 Days (*n* = 30)	Step 1	24	4	2	0
	Step 2—*Ad Hoc* model	17	6	5	2
	Step 2—Age-balanced model	15	5	4	6
	Step 3—*Ad Hoc* model	14	10	5	1
	Step 3—Age-balanced model	11	3	6	10

RD, Relative deviation.

### 3.2 Step 2—mucosal segmentation

Mucosal segmentation exhibited greater variability compared to Step 1. Relative deviations varied across age groups, with mean deviations as high as 24.6% in the youngest piglets but improving to 7.6–10.0% in older age groups (Table 1; Figure 2). The Pearson correlation coefficients dropped similarly, with an overall coefficient of 0.67 corresponding to strong linearity between AI-generated and manual annotations (Figure 3B, Supplementary Figure 1). Categorization of agreement level revealed a notable age-dependent trend in the performance of Step 2. For example, in the youngest group (4 days), only 10% of cases achieved “*very good*” agreement, while 40% were categorized as “*poor*.” Performance improved significantly in older groups, reaching 57% “*very good*” and only 7% “*poor*” at 67 days (Table 2). The spatial alignment of annotated areas mirrored the trend for the other performance parameters, yielding an overall IoU of 0.78 ± 0.19, and a positive association between IoU and increasing age (Table 1).

### 3.3 Step 3—epithelial segmentation

The final step in the segmentation model presented similar challenges as those in Step 2 with an overall mean deviation of 12.6%, and a large variation was seen between the different age groups ranging from 5.6–30.1%. As with the previous steps, the largest variation was seen in the youngest age group (4 days), which also affected the Pearson correlation coefficient for this age to drop to 0.49 (moderate linear correlation). The Pearson correlation coefficients for the remaining age groups were considerably stronger, varying from 0.77–0.96 (Table 1, Figure 3C; Supplementary Figure 2). When evaluating the categorized data, inferior performance was again observed in the youngest age groups, especially in the 4-day-old piglets, where more than half of the samples (55%) were assigned “*poor*.” The trend was almost opposite in the oldest age group, where 47% were categorized as “*very good*” agreement, and only 1 piglet ended up in the “*poor*” agreement category (Table 2). Despite the deviation in area size, the alignment between manually annotated and model-generated areas remained high, with an overall IoU of 0.92 ± 0.11.

### 3.4 Comparison of *Ad Hoc* and Age-balanced models

When comparing the performance of the *Ad Hoc* trained model to the Age-balanced model, notable differences emerged in Step 3. For Step 2 (mucosal segmentation), the overall performance was comparable between the two models (Table 1; Figures 2B, 3B). However, in Step 3 (epithelial segmentation), the *Ad Hoc* model consistently outperformed the Age-balanced model across all age groups. For example, in 49-day-old piglets, the *Ad Hoc* trained model achieved 59% “*very good*” agreement, while the Age-balanced model only achieved “*very good*” agreement in 7% of cases, and 70% was categorized as “*poor*” agreement (Table 2). Relative deviation, Pearson correlation coefficient, and IoU values mirrored these results, with the Age-balanced model exhibiting consistently larger deviations than the *Ad Hoc* model (Table 1; Figure 3C). The clear age-dependent decrease in relative deviation seen for the final step of the *Ad Hoc* trained model was not evident for the age-balanced model (Figure 2C).

### 3.5 Translatability to duodenum and ileum

The performance of the *Ad Hoc* model was evaluated on duodenum and ileum samples to assess its translatability across different segments of the small intestine. Tissue identification (Step 1) demonstrated consistent performance across all three anatomical regions (Supplementary Figure 3A). However, while mucosal segmentation (Step 2) showed poor results in the youngest age group for both duodenum and ileum, the performance was generally good in older age groups, with only slightly inferior performance in duodenum and ileum compared to the associated jejunum samples (Supplementary Figure 3B). Epithelial segmentation (Step 3) exhibited stable accuracy across all age groups in duodenum, comparable to jejunal performance. In contrast, ileum displayed an age-dependent improvement in accuracy, with better results in older piglets. Post-weaning, the relative deviation averaged −5% for duodenum, −4% for jejunum, and 0% for ileum, reflecting “*very good*” performance for Step 3 across all segments in older animals (Supplementary Figure 3C).

### 3.6 Qualitative assessment of model errors

During the comparison of AI-generated and manually annotated areas, a qualitative assessment of classifier errors was performed. Common segmentation errors were classification step-specific, although some mistakes were shared between Step 2 and 3 of the classification process.

#### 3.6.1 Step 1

**Figure 4:**
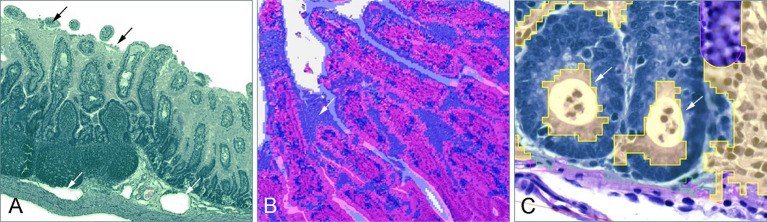
Frequently observed classification errors. **(A)** In step 1, luminal content and cellular debris was misclassified as part of the tissue (black arrows), and in cases of submucosal edema, dilated vessels were misinterpreted as part of the background (white arrow). **(B)** In step 2, fetal-type vacuolated epithelial cells on the villi of young piglets were often misclassified as part of the submucosa (white arrow). **(C)** In step 3, one of the commonly observed errors was the misclassification of crypt abscesses as part of the lamina propria (white arrows).

For Step 1, the classification of each pixel relied on a threshold value, which determined whether a pixel was categorized as background or tissue. While this approach was effective overall, several specific challenges were identified. In samples where the luminal content was not completely removed, or the lumen contained cellular debris, the classifier misinterpreted these elements as part of the tissue (Figure 4A). Moreover, due to the low-resolution settings of the classifier, small spaces between villi were often undetected and erroneously included in the tissue area. These false inclusions were more common in samples with tightly packed villi, especially prominent in younger animals. Finally, in piglets exhibiting substantial submucosal edema, dilated lymphatics and larger vessels were misclassified as background. The lack of staining in these regions caused the model to interpret them as non-tissue (Figure 4A).

### 3.7 Step 2

In Step 2, the segmentation of the tissue into mucosa and submucosa relied on the training of a random trees algorithm. A predominant error was observed in younger piglets (4–21 days), where vacuolated fetal-type enterocytes in the epithelial lining were frequently misclassified as part of the submucosal tissues due to their distinct texture and fainter staining intensity (Figure 4B).

#### 3.7.1 Step 3

In Step 3, pixel classification focused on differentiating the epithelial layer from the lamina propria, however, several recurring errors impacted model performance. Goblet cells, with their distinct cytoplasmic mucin stores and excentric, often not clearly visible nuclei, were occasionally misinterpreted as part of the lamina propria. Similarly, crypt abscesses, characterized by accumulations of cellular debris and neutrophilic granulocytes within crypt lumens, were often misclassified as lamina propria as well (Figure 4C). In younger piglets, the presence of fetal-type vacuolated epithelial cells, as seen in Step 2, contributed to a general overestimation of the lamina propria area by being incorrectly categorized as non-epithelial tissue. Conversely, in the age-balanced model, a notable underestimation of the lamina propria area was observed in piglets aged 25–67 days, where substantial portions of the lamina propria were misclassified as epithelial tissue, likely reflecting the model’s attempted adaptation to earlier developmental stages.

## 4 Discussion

This study demonstrated the feasibility of using a supervised ML model to divide the histological layers of the intestinal wall in piglets through a three-step segmentation approach. Despite being trained on only 8–15 samples—a sample size chosen to reflect the commonly available dataset sizes in veterinary studies and, also complying with the hardware limitations of a standard laptop—the model achieved “*good*” to “*very good*” agreement between AI-generated areas and manually annotated areas for a substantial proportion of the samples, particularly in piglets post-weaning. However, the structural complexity of the intestinal wall and the heterogeneity of the dataset suggest that additional training data would be required to enable a more unsupervised application of the model.

Surprisingly, the model showed reasonable translatability to other anatomical regions, including correct segmentation of structures like Brünner’s glands in the duodenum despite no prior training on this segment. This underlines the model’s potential applicability as a generalized “small intestine model,” rather than being specific to jejunal tissues, although tailored training for each anatomical location should always be preferred. Observed model errors fell into two main categories: (1) intrinsic limitations, such as the inability of the thresholding approach to distinguish between intestinal content and tissue, and (2) training deficits, such as misclassification of goblet cells and lesions like crypt abscesses. While the latter could most likely be resolved by including sufficient amounts of training examples, the intrinsic limitations are a bit different. For instance, in this study we applied a single-level thresholding method to identify tissue regions based on the optic density of each pixel. While effective for many samples, this method falls short in instances where images contain non-tissue components (e.g., intestinal content and staining artefacts) or unstained tissue elements (e.g., empty vascular structures). A way to overcome this issue would involve multilevel thresholding, using thresholds at different intensities or from multiple color channels (25). It should also be noted that the IoU values reported here are approximations rather than exact measurements, as QuPath relies on Java’s geometric operations (java.awt.geom.area). For highly irregular structures such as villi, or annotations with multiple holes, minor deviations can arise due to floating-point rounding or topological inconsistencies. Although generally small, these potential inaccuracies should be considered when interpreting IoU values in complex biological tissues. Furthermore, the commonly used object detection threshold of 0.50 may be insufficient for area-based annotations (26). In applications such as cell detection or counting, higher IoU levels may be necessary to ensure accurate quantitative results.

An additional consideration relates to the annotation process used for model training. In this study, all manual annotations were performed by a single observer. While this approach ensured consistency throughout the dataset, it introduces a potential observer bias. However, given that the annotated structures—such as mucosa, submucosa, and epithelial layers—are visually distinct and histologically well-defined, the impact of individual interpretation is expected to be minimal. Future studies could validate annotation robustness by incorporating interobserver variability assessments or consensus-based annotations from multiple pathologists.

A key finding was the age-related variation in model performance. Neonatal tissues, such as those containing fetal-type vacuolated epithelial cells, pose significant challenges for both pathologists and AI-models due to their unique morphology (27–30). Fetal-type vacuolated epithelial cells in the intestine are specialized cells, essential for the uptake of colostral macromolecules, and are gradually replaced by mature enterocytes by 3 weeks of age (31). Despite efforts to account for this by deliberately incorporating these variations into the Age-balanced model, it underperformed compared to the *Ad Hoc* model—particularly in older age groups-contrary to our expectations. This indicates that even the Age-balanced model struggled to accommodate the marked heterogeneity associated with gut development. The findings underscore the difficulty of achieving a reliable “one-size-fits-all” solution when working with age-diverse datasets. This challenge is not unique to the intestinal tract and is likely relevant for other organ systems with distinct neonatal morphology, such as the liver, kidneys, and brain. Researchers applying machine learning models to neonatal tissues should be aware of the intrinsic heterogeneity arising from tissue immaturity and consider this during both study design and model evaluation. Moreover, when using pretrained models, it is essential to verify the age range of the animals included in the original training data, as mismatched developmental stages may significantly compromise model performance. To enhance model robustness, future strategies could focus on either (1) expanding the training dataset to better capture biological variability, or (2) developing separate models for neonatal and mature tissues to reduce variability within datasets, while carefully managing the risk of overfitting. Furthermore, given the anatomical and physiological similarities between porcine and human tissues (32), incorporating transfer learning could also possibly leverage publicly available human datasets to augment training data. This approach would enable the model to benefit from larger datasets while retaining a focus on pig-specific features, addressing the challenges of limited sample sizes and supporting cross-species applicability.

From a disease perspective, the histopathological presentation of PWD in pigs is highly variable, and previous studies have failed to consistently associate specific lesions with the occurrence of clinical diarrhea, likely reflecting its multifactorial etiology (6, 7). Reported lesions include villous atrophy and fusion, inflammatory cell infiltrations, crypt abscesses, edema, hemorrhage, hyperemia, crypt hyperplasia and dilation, necrosis, and various types of exudations. From the perspective of the present segmentation model, some of these features are expected to be accommodated without difficulty, whereas others are likely to compromise performance. For example, the model is inherently resilient to changes such as villous atrophy, inflammatory infiltrations, edema, hyperemia, hemorrhage, and crypt hyperplasia, since these do not substantially alter the overall boundaries between histological layers and the individual cell morphology. In contrast, lesions that markedly alter surface architecture or introduce luminal structures not sufficiently represented in the training data—e.g. crypt abscesses, fibrinous exudation, pseudomembrane formation, and necrosis—are prone to misclassification. This was already evident in the present study for crypt abscesses, which were frequently misidentified, and similar limitations can be anticipated for other intraluminal or surface-associated lesions. Understanding these lesion-specific vulnerabilities is important when applying the model in studies of PWD or other enteropathies, as histopathological variability may influence segmentation accuracy and, consequently, quantitative outputs.

In conclusion, this study demonstrates that ML applications in histopathology are feasible even with small sample sizes, offering a practical and accessible tool for veterinary pathologists. However, models trained on limited datasets should be applied cautiously and not relied upon blindly. Users must remain highly aware of age-related morphological changes, which can significantly impact model training and performance. Instead, such models are best utilized as supervised/assisting tools to aid in generating quantitative measures, enabling pathologists to combine computational power with expert oversight.

## Data Availability

The raw data supporting the conclusions of this article will be made available by the authors, without undue reservation.
