# Supplementary material for: Machine-learning for quantitative histopathology of piglet intestinal tissues: challenges with limited training data
Source: Front Vet Sci. 2025 Oct 6;12:1620338. doi: 10.3389/fvets.2025.1620338 (PMC12536243; doi:10.3389/fvets.2025.1620338)
Supplement: Supplementary file 1 [file Supplementary_file_1.docx]

Supplementary Material


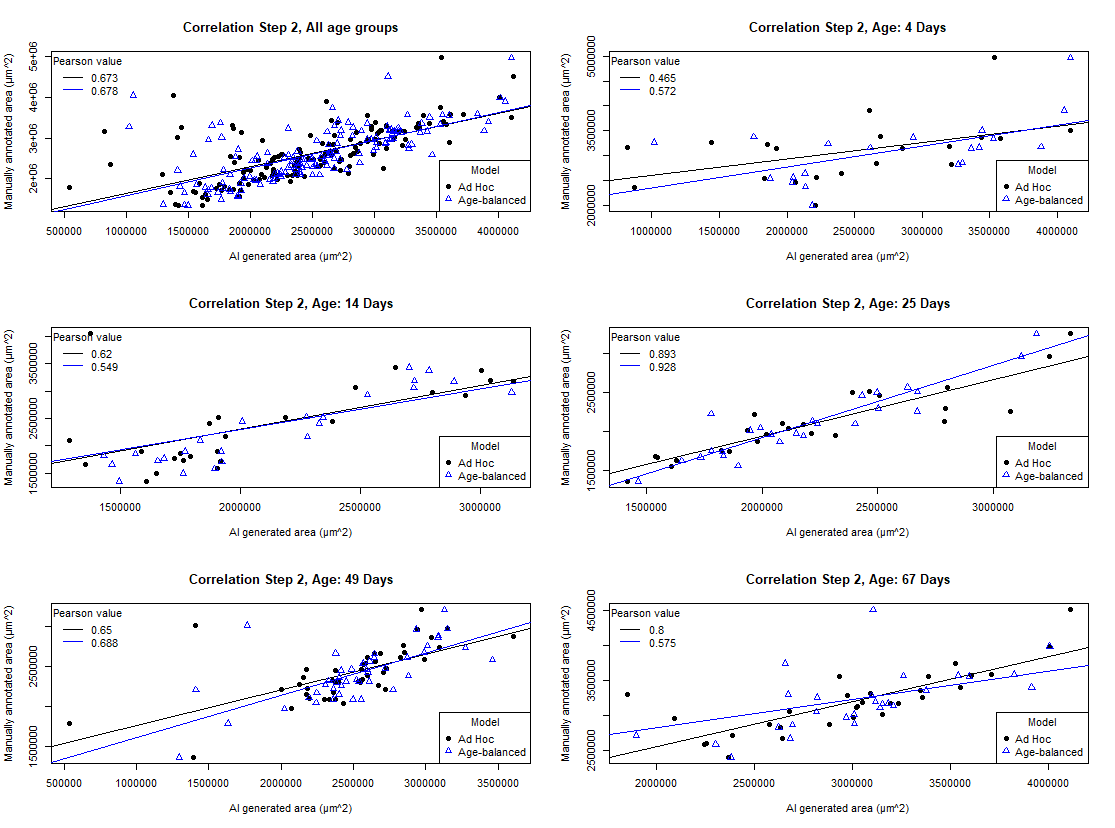


**Supplementary Figure 1.** Scatter plots of the correlation between AI-generated and manually annotated areas for mucosal segmentation (step 2) stratified according to age-group, with individual calculations of the Pearson correlation coefficient. Black dots represent the Ad Hoc trained model, whereas the blue triangles represent the Age-balanced model. Although the overall performance of the two models appears similar, a marked difference in PCC value between models is seen at day 67.

**
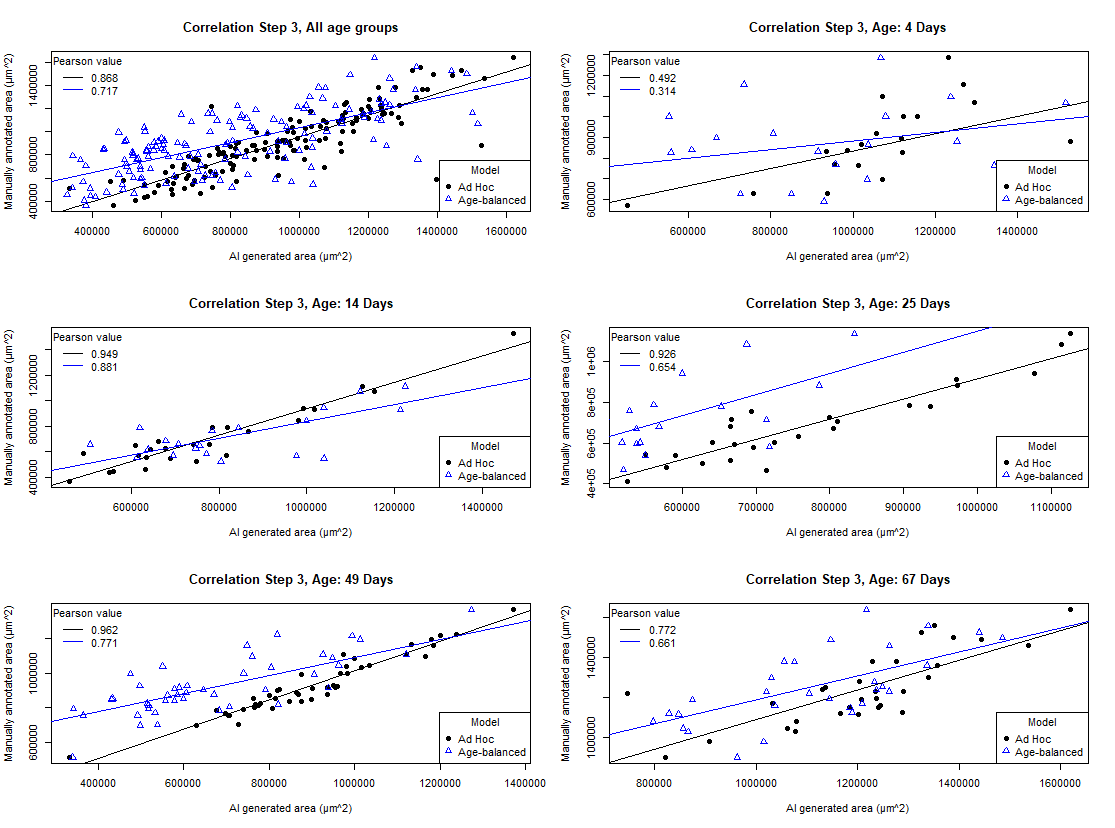
**

**Supplementary Figure 2.** Scatter plots of the correlation between AI-generated and manually annotated areas for epithelial segmentation (step 3) stratified according to age-group, with individual calculations of the Pearson correlation coefficient. Black dots represent the Ad Hoc trained model, whereas the blue triangles represent the Age-balanced model. At 4 days of age, the data seems to be almost randomly distributed for both models, with a very poor correlation coefficient, but in the remaining age groups, a clear linear correlation exists and is particularly evident for the Ad Hoc model.


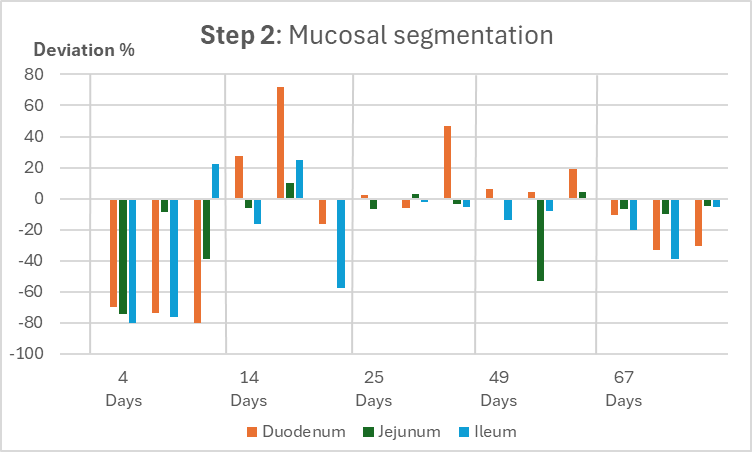

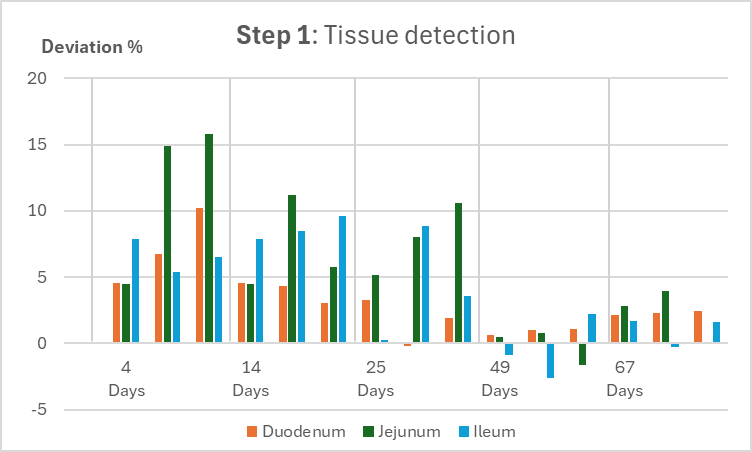

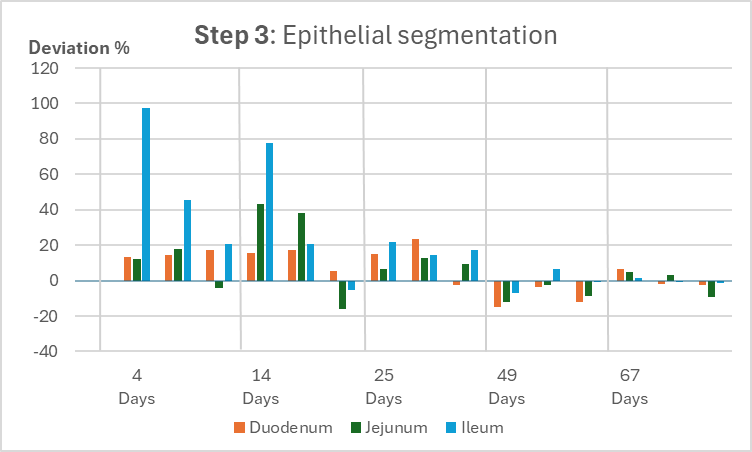


**A**

**B**

**C**

**Supplementary Figure 3.** Assessment of the transferability of the Ad Hoc model across different small intestinal segments by comparison of the relative deviation between AI-generated and manually annotated areas from three piglets of each age group. **A)** Performance in tissue detection (step 1) yielded consistent performance across all segments. **B)** Performance in step 2 – Mucosal segmentation. The performance of the model in duodenum and ileum is generally slightly inferior to that in the jejunum with marked age-dependent variation. **C)** Performance in step 3 – Epithelial segmentation. There is a clear increased performance with increasing age for all segments.

Script used for calculating Intersection over Union in QuPath:

import qupath.lib.roi.ROIs

import qupath.lib.geom.Point2

// Get exactly two selected annotations

def annotations = getSelectedObjects().findAll { it.getROI() != null }

if (annotations.size() != 2) {

print("Please select exactly TWO annotations!")

return

}

def ann1 = annotations[0]

def ann2 = annotations[1]

def roi1 = ann1.getROI()

def roi2 = ann2.getROI()

// Convert ROIs to java.awt.geom.Area objects

def area1 = new java.awt.geom.Area(roi1.getShape())

def area2 = new java.awt.geom.Area(roi2.getShape())

// Calculate intersection and union

def intersection = new java.awt.geom.Area(area1)

intersection.intersect(area2)

def union = new java.awt.geom.Area(area1)

union.add(area2)

// Function to convert java.awt.geom.Area to QuPath ROI

def areaToROI(java.awt.geom.Area area) {

def path = area.getPathIterator(null)

def coords = new double[6]

def points = []

while (!path.isDone()) {

int type = path.currentSegment(coords)

if (type == java.awt.geom.PathIterator.SEG_MOVETO || type == java.awt.geom.PathIterator.SEG_LINETO) {

points << new Point2(coords[0], coords[1])

}

path.next()

}

if (points.size() < 3) {

return null // Not a valid polygon

}

return ROIs.createPolygonROI(points, null)

}

// Convert intersection and union areas back to ROI

def intersectionROI = areaToROI(intersection)

def unionROI = areaToROI(union)

if (intersectionROI == null || unionROI == null) {

print("Could not create valid ROI from intersection or union shapes. IoU calculation aborted.")

return

}

// Calculate areas using QuPath ROI's getArea() method

def intersectionArea = intersectionROI.getArea()

def unionArea = unionROI.getArea()

// Calculate IoU

def iou = 0

if (unionArea > 0) {

iou = intersectionArea / unionArea

}

println "Intersection area: " + intersectionArea

println "Union area: " + unionArea

println "Intersection over Union (IoU): " + iou

R-code for statistical analysis:

# Loading of data into R:

setwd("H:/From_SUND/PhD PIG-PARADIGM/Publikationer/Machine Learning for Quantitative Histopathology of Piglet Intestinal Lesions/Statistik")

library(readxl)

Area_Data <- read_excel("Area_Data.xlsx")

# Create vectors of relative deviation:

Area_Data$Dev_1 <- (((Area_Data$AI_tissue-Area_Data$Manual_tissue)/Area_Data$Manual_tissue)*100)

Area_Data$Dev_2AdHoc <- (((Area_Data$AI_mucosa_AdHoc-Area_Data$Manual_mucosa)/Area_Data$Manual_mucosa)*100)

Area_Data$Dev_2AgeStrat <- (((Area_Data$AI_mucosa_AgeStrat-Area_Data$Manual_mucosa)/Area_Data$Manual_mucosa)*100)

Area_Data$Dev_3AdHoc <- (((Area_Data$AI_LP_AdHoc-Area_Data$Manual_LP)/Area_Data$Manual_LP)*100)

Area_Data$Dev_3AgeStrat <- (((Area_Data$AI_LP_AgeStrat-Area_Data$Manual_LP)/Area_Data$Manual_LP)*100)

# Testing data for normality using a Shapiro Wilks test and visualized by histograms:

library("dplyr")

par(mfrow=c(3,3))

shapiro.test(Area_Data$Manual_tissue)

hist(Area_Data$Manual_tissue, main="Manual Step 1", col="darkgreen", xlab="Tissue area")

shapiro.test(Area_Data$AI_tissue)

hist(Area_Data$AI_tissue, main="AI Step 1", col="darkgreen", xlab="Tissue area")

shapiro.test(Area_Data$Manual_mucosa)

hist(Area_Data$Manual_mucosa, main="Manual Step 2", col="magenta", xlab="Mucosal area")

shapiro.test(Area_Data$AI_mucosa_AdHoc)

hist(Area_Data$AI_mucosa_AdHoc, main="AI Step 2 AdHoc", col="magenta", xlab="Mucosal area")

shapiro.test(Area_Data$AI_mucosa_AgeStrat)

hist(Area_Data$AI_mucosa_AgeStrat, main="AI Step 2 AgeStrat", col="magenta", xlab="Mucosal area")

shapiro.test(Area_Data$Manual_LP)

hist(Area_Data$Manual_LP, main="Manual Step 3", col="purple3", xlab="Lamina propria area")

shapiro.test(Area_Data$AI_LP_AdHoc)

hist(Area_Data$AI_LP_AdHoc, main="AI Step 3 AdHoc", col="purple3", xlab="Lamina propria area")

shapiro.test(Area_Data$AI_LP_AgeStrat)

hist(Area_Data$AI_LP_AgeStrat, main="AI Step 3 AgeStrat", col="purple3", xlab="Lamina propria area")

shapiro.test(Area_Data$Dev_1)

hist(Area_Data$Dev_1, main="Relative deviation Step 1", col="darkgreen", xlab="RD step 1")

shapiro.test(Area_Data$Dev_2AgeStrat)

hist(Area_Data$Dev_2AgeStrat, main="Relative deviation Step 2 AgeStrat", col="magenta", xlab="RD step 2")

shapiro.test(Area_Data$Dev_2AdHoc)

hist(Area_Data$Dev_2AdHoc, main="Relative deviation Step 2 AdHoc", col="magenta", xlab="RD step 2")

shapiro.test(Area_Data$Dev_3AgeStrat)

hist(Area_Data$Dev_3AgeStrat, main="Relative deviation Step 3 AgeStrat", col="purple3", xlab="RD step 3")

shapiro.test(Area_Data$Dev_3AdHoc)

hist(Area_Data$Dev_3AdHoc, main="Relative deviation Step 3 AdHoc", col="purple3", xlab="RD step 3")

# Subsetting data according to age groups:

Area_Data4 <- subset(Area_Data, Age==4)

Area_Data14 <- subset(Area_Data, Age==14)

Area_Data25 <- subset(Area_Data, Age==25)

Area_Data49 <- subset(Area_Data, Age==49)

Area_Data67 <- subset(Area_Data, Age==67)

# Scatterplot and Pearson correlation of step 1 (tissue identification)

cor.test(Area_Data$Manual_tissue,Area_Data$AI_tissue,method="pearson")

plot(Area_Data$AI_tissue,Area_Data$Manual_tissue,xlab="AI generated area (µm^2)",ylab="Manually annotated area (µm^2)",main="Correlation in Step 1, Tissue detection", pch=19, cex.lab=1.2, cex.axis=1.2, cex.main=1.5)

abline(lm(Area_Data$Manual_tissue~Area_Data$AI_tissue))

legend("bottomright",legend="Tissue identification",pch=19,col="black")

legend("topleft",legend=0.968, title="Pearson value", lty=1, col="black", bty="n",cex=1.3)

#Pearson correlation of step 1 divided into age groups:

cor.test(Area_Data4$Manual_tissue,Area_Data4$AI_tissue,method="pearson")

cor.test(Area_Data14$Manual_tissue,Area_Data14$AI_tissue,method="pearson")

cor.test(Area_Data25$Manual_tissue,Area_Data25$AI_tissue,method="pearson")

cor.test(Area_Data49$Manual_tissue,Area_Data49$AI_tissue,method="pearson")

cor.test(Area_Data67$Manual_tissue,Area_Data67$AI_tissue,method="pearson")

# Scatterplot and pearson correlation of step 2 (mucosa identification) for all age groups with comparison of the two models:

cor.test(Area_Data$Manual_mucosa,Area_Data$AI_mucosa_AdHoc,method="pearson")

cor.test(Area_Data$Manual_mucosa,Area_Data$AI_mucosa_AgeStrat,method="pearson")

plot(Area_Data$AI_mucosa_AdHoc,Area_Data$Manual_mucosa,xlab="AI generated area (µm^2)",ylab="Manually annotated area (µm^2)",main="Correlation Step 2, All age groups", pch=19)

abline(lm(Area_Data$Manual_mucosa~Area_Data$AI_mucosa_AdHoc))

points(Area_Data$AI_mucosa_AgeStrat,Area_Data$Manual_mucosa,pch=2,col="blue")

abline(lm(Area_Data$Manual_mucosa~Area_Data$AI_mucosa_AgeStrat),col="blue")

legend("bottomright",legend=c("Ad Hoc","Age-balanced"),pch=c(19,2),col=c("black","blue"), title="Model",cex=1)

legend("topleft",legend=c(0.673,0.678), title="Pearson value", lty=1, col=c("black","blue"), bty="n", cex=1)

#Scatterplot and pearson correlation of step 3 (mucosa segmentation) for all age groups with comparison of the two models:

cor.test(Area_Data$Manual_LP,Area_Data$AI_LP_AdHoc,method="pearson")

cor.test(Area_Data$Manual_LP,Area_Data$AI_LP_AgeStrat,method="pearson")

plot(Area_Data$AI_LP_AdHoc,Area_Data$Manual_LP,xlab="AI generated area (µm^2)",ylab="Manually annotated area (µm^2)",main="Correlation Step 3, All age groups", pch=19)

abline(lm(Area_Data$Manual_LP~Area_Data$AI_LP_AdHoc))

points(Area_Data$AI_LP_AgeStrat,Area_Data$Manual_LP,pch=2,col="blue")

abline(lm(Area_Data$Manual_LP~Area_Data$AI_LP_AgeStrat),col="blue")

legend("bottomright",legend=c("Ad Hoc","Age-balanced"),pch=c(19,2),col=c("black","blue"), title="Model", cex=1)

legend("topleft",legend=c(0.868,0.717), title="Pearson value", lty=1, col=c("black","blue"), bty="n", cex=1)

# Scatterplot and pearson correlation of step 2, divided into age groups for both models:

# Age:4 Days

cor.test(Area_Data4$Manual_mucosa,Area_Data4$AI_mucosa_AdHoc,method="pearson")

cor.test(Area_Data4$Manual_mucosa,Area_Data4$AI_mucosa_AgeStrat,method="pearson")

plot(Area_Data4$AI_mucosa_AdHoc,Area_Data4$Manual_mucosa,xlab="AI generated area (µm^2)",ylab="Manually annotated area (µm^2)",main="Correlation Step 2, Age: 4 Days", pch=19)

abline(lm(Area_Data4$Manual_mucosa~Area_Data4$AI_mucosa_AdHoc))

points(Area_Data4$AI_mucosa_AgeStrat,Area_Data4$Manual_mucosa,pch=2,col="blue")

abline(lm(Area_Data4$Manual_mucosa~Area_Data4$AI_mucosa_AgeStrat),col="blue")

legend("bottomright",legend=c("Ad Hoc","Age-balanced"),pch=c(19,2),col=c("black","blue"), title="Model")

legend("topleft",legend=c(0.465,0.572), title="Pearson value", lty=1, col=c("black","blue"), bty="n")

# Age:14 Days

cor.test(Area_Data14$Manual_mucosa,Area_Data14$AI_mucosa_AdHoc,method="pearson")

cor.test(Area_Data14$Manual_mucosa,Area_Data14$AI_mucosa_AgeStrat,method="pearson")

plot(Area_Data14$AI_mucosa_AdHoc,Area_Data14$Manual_mucosa,xlab="AI generated area (µm^2)",ylab="Manually annotated area (µm^2)",main="Correlation Step 2, Age: 14 Days", pch=19)

abline(lm(Area_Data14$Manual_mucosa~Area_Data14$AI_mucosa_AdHoc))

points(Area_Data14$AI_mucosa_AgeStrat,Area_Data14$Manual_mucosa,pch=2,col="blue")

abline(lm(Area_Data14$Manual_mucosa~Area_Data14$AI_mucosa_AgeStrat),col="blue")

legend("bottomright",legend=c("Ad Hoc","Age-balanced"),pch=c(19,2),col=c("black","blue"), title="Model")

legend("topleft",legend=c(0.620,0.549), title="Pearson value", lty=1, col=c("black","blue"), bty="n")

# Age:25 Days

cor.test(Area_Data25$Manual_mucosa,Area_Data25$AI_mucosa_AdHoc,method="pearson")

cor.test(Area_Data25$Manual_mucosa,Area_Data25$AI_mucosa_AgeStrat,method="pearson")

plot(Area_Data25$AI_mucosa_AdHoc,Area_Data25$Manual_mucosa,xlab="AI generated area (µm^2)",ylab="Manually annotated area (µm^2)",main="Correlation Step 2, Age: 25 Days", pch=19)

abline(lm(Area_Data25$Manual_mucosa~Area_Data25$AI_mucosa_AdHoc))

points(Area_Data25$AI_mucosa_AgeStrat,Area_Data25$Manual_mucosa,pch=2,col="blue")

abline(lm(Area_Data25$Manual_mucosa~Area_Data25$AI_mucosa_AgeStrat),col="blue")

legend("bottomright",legend=c("Ad Hoc","Age-balanced"),pch=c(19,2),col=c("black","blue"), title="Model")

legend("topleft",legend=c(0.893,0.928), title="Pearson value", lty=1, col=c("black","blue"), bty="n")

#Age:49 Days

cor.test(Area_Data49$Manual_mucosa,Area_Data49$AI_mucosa_AdHoc,method="pearson")

cor.test(Area_Data49$Manual_mucosa,Area_Data49$AI_mucosa_AgeStrat,method="pearson")

plot(Area_Data49$AI_mucosa_AdHoc,Area_Data49$Manual_mucosa,xlab="AI generated area (µm^2)",ylab="Manually annotated area (µm^2)",main="Correlation Step 2, Age: 49 Days", pch=19)

abline(lm(Area_Data49$Manual_mucosa~Area_Data49$AI_mucosa_AdHoc))

points(Area_Data49$AI_mucosa_AgeStrat,Area_Data49$Manual_mucosa,pch=2,col="blue")

abline(lm(Area_Data49$Manual_mucosa~Area_Data49$AI_mucosa_AgeStrat),col="blue")

legend("bottomright",legend=c("Ad Hoc","Age-balanced"),pch=c(19,2),col=c("black","blue"), title="Model")

legend("topleft",legend=c(0.650,0.688), title="Pearson value", lty=1, col=c("black","blue"), bty="n")

#Age:67 Days

cor.test(Area_Data67$Manual_mucosa,Area_Data67$AI_mucosa_AdHoc,method="pearson")

cor.test(Area_Data67$Manual_mucosa,Area_Data67$AI_mucosa_AgeStrat,method="pearson")

plot(Area_Data67$AI_mucosa_AdHoc,Area_Data67$Manual_mucosa,xlab="AI generated area (µm^2)",ylab="Manually annotated area (µm^2)",main="Correlation Step 2, Age: 67 Days", pch=19)

abline(lm(Area_Data67$Manual_mucosa~Area_Data67$AI_mucosa_AdHoc))

points(Area_Data67$AI_mucosa_AgeStrat,Area_Data67$Manual_mucosa,pch=2,col="blue")

abline(lm(Area_Data67$Manual_mucosa~Area_Data67$AI_mucosa_AgeStrat),col="blue")

legend("bottomright",legend=c("Ad Hoc","Age-balanced"),pch=c(19,2),col=c("black","blue"), title="Model")

legend("topleft",legend=c(0.800,0.575), title="Pearson value", lty=1, col=c("black","blue"), bty="n")

# Scatterplot and pearson correlation of step 3, divided into age groups for both models:

# Age:4 Days

cor.test(Area_Data4$Manual_LP,Area_Data4$AI_LP_AdHoc,method="pearson")

cor.test(Area_Data4$Manual_LP,Area_Data4$AI_LP_AgeStrat,method="pearson")

plot(Area_Data4$AI_LP_AdHoc,Area_Data4$Manual_LP,xlab="AI generated area (µm^2)",ylab="Manually annotated area (µm^2)",main="Correlation Step 3, Age: 4 Days", pch=19)

abline(lm(Area_Data4$Manual_LP~Area_Data4$AI_LP_AdHoc))

points(Area_Data4$AI_LP_AgeStrat,Area_Data4$Manual_LP,pch=2,col="blue")

abline(lm(Area_Data4$Manual_LP~Area_Data4$AI_LP_AgeStrat),col="blue")

legend("bottomright",legend=c("Ad Hoc","Age-balanced"),pch=c(19,2),col=c("black","blue"), title="Model")

legend("topleft",legend=c(0.492,0.314), title="Pearson value", lty=1, col=c("black","blue"), bty="n")

# Age:14 Days

cor.test(Area_Data14$Manual_LP,Area_Data14$AI_LP_AdHoc,method="pearson")

cor.test(Area_Data14$Manual_LP,Area_Data14$AI_LP_AgeStrat,method="pearson")

plot(Area_Data14$AI_LP_AdHoc,Area_Data14$Manual_LP,xlab="AI generated area (µm^2)",ylab="Manually annotated area (µm^2)",main="Correlation Step 3, Age: 14 Days", pch=19)

abline(lm(Area_Data14$Manual_LP~Area_Data14$AI_LP_AdHoc))

points(Area_Data14$AI_LP_AgeStrat,Area_Data14$Manual_LP,pch=2,col="blue")

abline(lm(Area_Data14$Manual_LP~Area_Data14$AI_LP_AgeStrat),col="blue")

legend("bottomright",legend=c("Ad Hoc","Age-balanced"),pch=c(19,2),col=c("black","blue"), title="Model")

legend("topleft",legend=c(0.949,0.881), title="Pearson value", lty=1, col=c("black","blue"), bty="n")

# Age:25 Days

cor.test(Area_Data25$Manual_LP,Area_Data25$AI_LP_AdHoc,method="pearson")

cor.test(Area_Data25$Manual_LP,Area_Data25$AI_LP_AgeStrat,method="pearson")

plot(Area_Data25$AI_LP_AdHoc,Area_Data25$Manual_LP,xlab="AI generated area (µm^2)",ylab="Manually annotated area (µm^2)",main="Correlation Step 3, Age: 25 Days", pch=19)

abline(lm(Area_Data25$Manual_LP~Area_Data25$AI_LP_AdHoc))

points(Area_Data25$AI_LP_AgeStrat,Area_Data25$Manual_LP,pch=2,col="blue")

abline(lm(Area_Data25$Manual_LP~Area_Data25$AI_LP_AgeStrat),col="blue")

legend("bottomright",legend=c("Ad Hoc","Age-balanced"),pch=c(19,2),col=c("black","blue"), title="Model")

legend("topleft",legend=c(0.926,0.654), title="Pearson value", lty=1, col=c("black","blue"), bty="n")

# Age:49 Days

cor.test(Area_Data49$Manual_LP,Area_Data49$AI_LP_AdHoc,method="pearson")

cor.test(Area_Data49$Manual_LP,Area_Data49$AI_LP_AgeStrat,method="pearson")

plot(Area_Data49$AI_LP_AdHoc,Area_Data49$Manual_LP,xlab="AI generated area (µm^2)",ylab="Manually annotated area (µm^2)",main="Correlation Step 3, Age: 49 Days", pch=19)

abline(lm(Area_Data49$Manual_LP~Area_Data49$AI_LP_AdHoc))

points(Area_Data49$AI_LP_AgeStrat,Area_Data49$Manual_LP,pch=2,col="blue")

abline(lm(Area_Data49$Manual_LP~Area_Data49$AI_LP_AgeStrat),col="blue")

legend("bottomright",legend=c("Ad Hoc","Age-balanced"),pch=c(19,2),col=c("black","blue"), title="Model")

legend("topleft",legend=c(0.962,0.771), title="Pearson value", lty=1, col=c("black","blue"), bty="n")

# Age:67 Days

cor.test(Area_Data67$Manual_LP,Area_Data67$AI_LP_AdHoc,method="pearson")

cor.test(Area_Data67$Manual_LP,Area_Data67$AI_LP_AgeStrat,method="pearson")

plot(Area_Data67$AI_LP_AdHoc,Area_Data67$Manual_LP,xlab="AI generated area (µm^2)",ylab="Manually annotated area (µm^2)",main="Correlation Step 3, Age: 67 Days", pch=19)

abline(lm(Area_Data67$Manual_LP~Area_Data67$AI_LP_AdHoc))

points(Area_Data67$AI_LP_AgeStrat,Area_Data67$Manual_LP,pch=2,col="blue")

abline(lm(Area_Data67$Manual_LP~Area_Data67$AI_LP_AgeStrat),col="blue")

legend("bottomright",legend=c("Ad Hoc","Age-balanced"),pch=c(19,2),col=c("black","blue"), title="Model")

legend("topleft",legend=c(0.772,0.661), title="Pearson value", lty=1, col=c("black","blue"), bty="n")

______________________________________________________________________________________________________

# Before calculating the min, max, median and mean, the deviations are converted to absolute values, since we are only interested in the size of the difference not the direction:

Area_Data$Dev_1abs <- abs(Area_Data$Dev_1)

Area_Data$Dev_2AdHoc_abs <- abs(Area_Data$Dev_2AdHoc)

Area_Data$Dev_2AgeStrat_abs <- abs(Area_Data$Dev_2AgeStrat)

Area_Data$Dev_3AdHoc_abs <- abs(Area_Data$Dev_3AdHoc)

Area_Data$Dev_3AgeStrat_abs <- abs(Area_Data$Dev_3AgeStrat)

# Summary statistics of absolute values - overall

summary(Area_Data$Dev_1abs)

sd(Area_Data$Dev_1abs)

summary(Area_Data$Dev_2AdHoc_abs)

sd(Area_Data$Dev_2AdHoc_abs)

summary(Area_Data$Dev_2AgeStrat_abs)

sd(Area_Data$Dev_2AgeStrat_abs)

summary(Area_Data$Dev_3AdHoc_abs)

sd(Area_Data$Dev_3AdHoc_abs)

summary(Area_Data$Dev_3AgeStrat_abs)

sd(Area_Data$Dev_3AgeStrat_abs)

# Summary statistics of absolute values - age groups

# 4 Days

summary(Area_Data4$Dev_1abs)

sd(Area_Data4$Dev_1abs)

summary(Area_Data4$Dev_2AdHoc_abs)

sd(Area_Data4$Dev_2AdHoc_abs)

summary(Area_Data4$Dev_2AgeStrat_abs)

sd(Area_Data4$Dev_2AgeStrat_abs)

summary(Area_Data4$Dev_3AdHoc_abs)

sd(Area_Data4$Dev_3AdHoc_abs)

summary(Area_Data4$Dev_3AgeStrat_abs)

sd(Area_Data4$Dev_3AgeStrat_abs)

# 14 Days

summary(Area_Data14$Dev_1abs)

sd(Area_Data14$Dev_1abs)

summary(Area_Data14$Dev_2AdHoc_abs)

sd(Area_Data14$Dev_2AdHoc_abs)

summary(Area_Data14$Dev_2AgeStrat_abs)

sd(Area_Data14$Dev_2AgeStrat_abs)

summary(Area_Data14$Dev_3AdHoc_abs)

sd(Area_Data14$Dev_3AdHoc_abs)

summary(Area_Data14$Dev_3AgeStrat_abs)

sd(Area_Data14$Dev_3AgeStrat_abs)

# 25 Days

summary(Area_Data25$Dev_1abs)

sd(Area_Data25$Dev_1abs)

summary(Area_Data25$Dev_2AdHoc_abs)

sd(Area_Data25$Dev_2AdHoc_abs)

summary(Area_Data25$Dev_2AgeStrat_abs)

sd(Area_Data25$Dev_2AgeStrat_abs)

summary(Area_Data25$Dev_3AdHoc_abs)

sd(Area_Data25$Dev_3AdHoc_abs)

summary(Area_Data25$Dev_3AgeStrat_abs)

sd(Area_Data25$Dev_3AgeStrat_abs)

# 49 Days

summary(Area_Data49$Dev_1abs)

sd(Area_Data49$Dev_1abs)

summary(Area_Data49$Dev_2AdHoc_abs)

sd(Area_Data49$Dev_2AdHoc_abs)

summary(Area_Data49$Dev_2AgeStrat_abs)

sd(Area_Data49$Dev_2AgeStrat_abs)

summary(Area_Data49$Dev_3AdHoc_abs)

sd(Area_Data49$Dev_3AdHoc_abs)

summary(Area_Data49$Dev_3AgeStrat_abs)

sd(Area_Data49$Dev_3AgeStrat_abs)

# 67 Days

summary(Area_Data67$Dev_1abs)

sd(Area_Data67$Dev_1abs)

summary(Area_Data67$Dev_2AdHoc_abs)

sd(Area_Data67$Dev_2AdHoc_abs)

summary(Area_Data67$Dev_2AgeStrat_abs)

sd(Area_Data67$Dev_2AgeStrat_abs)

summary(Area_Data67$Dev_3AdHoc_abs)

sd(Area_Data67$Dev_3AdHoc_abs)

summary(Area_Data67$Dev_3AgeStrat_abs)

sd(Area_Data67$Dev_3AgeStrat_abs)

# Create a modified Bland-Altman plot

par(mfrow=c(3,2))

#Step 1:

plot(Area_Data4$Number,Area_Data4$Dev_1,xlab="Age (Days)",ylab="Relative deviation (%)",type="h",col="orange",xlim=c(0,145),ylim=c(-30,30),xaxt="n",las=1,main="Relative deviation - Step 1",cex.lab=1.3,cex.main=1.5, cex.axis=1.3)

axis(1,at=c(10,32,57,92,130),labels=c(4,14,25,49,67),cex.axis=1.3)

points(Area_Data14$Number,Area_Data14$Dev_1, col="darkgreen",type="h")

points(Area_Data25$Number,Area_Data25$Dev_1, col="black",type="h")

points(Area_Data49$Number,Area_Data49$Dev_1, col="darkblue",type="h")

points(Area_Data67$Number,Area_Data67$Dev_1, col="red4",type="h")

abline(h=0,col="black",lty=1)

abline(v=20,col="grey",lty=3)

abline(v=45,col="grey",lty=3)

abline(v=70,col="grey",lty=3)

abline(v=115,col="grey",lty=3)

#Step 2AdHoc trained model:

summary(Area_Data$Dev_2AdHoc)

plot(Area_Data4$Number,Area_Data4$Dev_2AdHoc,xlab="Age (Days)",ylab="Relative deviation (%)",type="h",col="orange",xlim=c(0,145),ylim=c(-75,75),xaxt="n",las=1,main="Relative deviation - Step 2 Ad Hoc")

axis(1,at=c(10,32,57,92,130),labels=c(4,14,25,49,67))

points(Area_Data14$Number,Area_Data14$Dev_2AdHoc, col="darkgreen",type="h")

points(Area_Data25$Number,Area_Data25$Dev_2AdHoc, col="black",type="h")

points(Area_Data49$Number,Area_Data49$Dev_2AdHoc, col="darkblue",type="h")

points(Area_Data67$Number,Area_Data67$Dev_2AdHoc, col="red4",type="h")

abline(h=0,col="black",lty=1)

abline(v=20,col="grey",lty=3)

abline(v=45,col="grey",lty=3)

abline(v=70,col="grey",lty=3)

abline(v=115,col="grey",lty=3)

#Step 3 AdHoc trained model:

summary(Area_Data$Dev_3AdHoc)

plot(Area_Data4$Number,Area_Data4$Dev_3AdHoc,xlab="Age (Days)",ylab="Relative deviation (%)",type="h",col="orange",xlim=c(0,145),ylim=c(-40,100),xaxt="n",las=1,main="Relative deviation - Step 3 Ad Hoc")

axis(1,at=c(10,32,57,92,130),labels=c(4,14,25,49,67))

points(Area_Data14$Number,Area_Data14$Dev_3AdHoc, col="darkgreen",type="h")

points(Area_Data25$Number,Area_Data25$Dev_3AdHoc, col="black",type="h")

points(Area_Data49$Number,Area_Data49$Dev_3AdHoc, col="darkblue",type="h")

points(Area_Data67$Number,Area_Data67$Dev_3AdHoc, col="red4",type="h")

abline(h=0,col="black",lty=1)

abline(v=20,col="grey",lty=3)

abline(v=45,col="grey",lty=3)

abline(v=70,col="grey",lty=3)

abline(v=115,col="grey",lty=3)

# Step 2 Age stratified model:

summary(Area_Data$Dev_2AgeStrat)

plot(Area_Data4$Number,Area_Data4$Dev_2AgeStrat,xlab="Age (Days)",ylab="Relative deviation (%)",type="h",col="orange",xlim=c(0,145),ylim=c(-75,75),xaxt="n",las=1,main="Relative deviation - Step 2 Age stratified")

axis(1,at=c(10,32,57,92,130),labels=c(4,14,25,49,67))

points(Area_Data14$Number,Area_Data14$Dev_2AgeStrat, col="darkgreen",type="h")

points(Area_Data25$Number,Area_Data25$Dev_2AgeStrat, col="black",type="h")

points(Area_Data49$Number,Area_Data49$Dev_2AgeStrat, col="darkblue",type="h")

points(Area_Data67$Number,Area_Data67$Dev_2AgeStrat, col="red4",type="h")

abline(h=0,col="black",lty=1)

abline(v=20,col="grey",lty=3)

abline(v=45,col="grey",lty=3)

abline(v=70,col="grey",lty=3)

abline(v=115,col="grey",lty=3)

# Step 3 Age stratified model:

summary(Area_Data$Dev_3AgeStrat)

plot(Area_Data4$Number,Area_Data4$Dev_3AgeStrat,xlab="Age (Days)",ylab="Relative deviation (%)",type="h",col="orange",xlim=c(0,145),ylim=c(-75,90),xaxt="n",las=1,main="Relative deviation - Step 3 Age stratified")

axis(1,at=c(10,32,57,92,130),labels=c(4,14,25,49,67))

points(Area_Data14$Number,Area_Data14$Dev_3AgeStrat, col="darkgreen",type="h")

points(Area_Data25$Number,Area_Data25$Dev_3AgeStrat, col="black",type="h")

points(Area_Data49$Number,Area_Data49$Dev_3AgeStrat, col="darkblue",type="h")

points(Area_Data67$Number,Area_Data67$Dev_3AgeStrat, col="red4",type="h")

abline(h=0,col="black",lty=1)

abline(v=20,col="grey",lty=3)

abline(v=45,col="grey",lty=3)

abline(v=70,col="grey",lty=3)

abline(v=115,col="grey",lty=3)

# Comparison of AdHoc and Age stratified model on the same plot:

# Creation of semitransparent color for the plot:

col2rgb("orange")

MyCol <- rgb(255,165,0, max=255, alpha=175, names="orange50")

#Step 2:

plot(Area_Data4$Number,Area_Data4$Dev_2AdHoc,xlab="Age (Days)",ylab="Relative deviation (%)",type="h",col="black",xlim=c(0,145),ylim=c(-80,80),xaxt="n",las=1,main="Relative deviation - Step 2, comparison",cex.lab=1.3,cex.main=1.5, cex.axis=1.3)

axis(1,at=c(10,32,57,92,130),labels=c(4,14,25,49,67),cex.axis=1.3)

points(Area_Data14$Number,Area_Data14$Dev_2AdHoc, col="black",type="h")

points(Area_Data25$Number,Area_Data25$Dev_2AdHoc, col="black",type="h")

points(Area_Data49$Number,Area_Data49$Dev_2AdHoc, col="black",type="h")

points(Area_Data67$Number,Area_Data67$Dev_2AdHoc, col="black",type="h")

abline(h=0,col="black",lty=1)

abline(v=20,col="grey",lty=3)

abline(v=45,col="grey",lty=3)

abline(v=70,col="grey",lty=3)

abline(v=115,col="grey",lty=3)

points(Area_Data4$Number,Area_Data4$Dev_2AgeStrat,col="#FFA5007D",type="h")

points(Area_Data14$Number,Area_Data14$Dev_2AgeStrat, col="#FFA5007D",type="h")

points(Area_Data25$Number,Area_Data25$Dev_2AgeStrat, col="#FFA5007D",type="h")

points(Area_Data49$Number,Area_Data49$Dev_2AgeStrat, col="#FFA5007D",type="h")

points(Area_Data67$Number,Area_Data67$Dev_2AgeStrat, col="#FFA5007D",type="h")

legend("topright",lty=1,col=c("black","#FFA5007D"),legend=c("Ad Hoc","Age-balanced"),title="Model",cex=1.1,bty="n")

#Step 3:

plot(Area_Data4$Number,Area_Data4$Dev_3AdHoc,xlab="Age (Days)",ylab="Relative deviation (%)",type="h",col="black",xlim=c(0,145),ylim=c(-100,100),xaxt="n",las=1,main="Relative deviation - Step 3, comparison",cex.lab=1.3,cex.main=1.5, cex.axis=1.3)

axis(1,at=c(10,32,57,92,130),labels=c(4,14,25,49,67),cex.axis=1.3)

points(Area_Data14$Number,Area_Data14$Dev_3AdHoc, col="black",type="h")

points(Area_Data25$Number,Area_Data25$Dev_3AdHoc, col="black",type="h")

points(Area_Data49$Number,Area_Data49$Dev_3AdHoc, col="black",type="h")

points(Area_Data67$Number,Area_Data67$Dev_3AdHoc, col="black",type="h")

abline(h=0,col="black",lty=1)

abline(v=20,col="grey",lty=3)

abline(v=45,col="grey",lty=3)

abline(v=70,col="grey",lty=3)

abline(v=115,col="grey",lty=3)

points(Area_Data4$Number,Area_Data4$Dev_3AgeStrat,col="#FFA5007D",type="h")

points(Area_Data14$Number,Area_Data14$Dev_3AgeStrat, col="#FFA5007D",type="h")

points(Area_Data25$Number,Area_Data25$Dev_3AgeStrat, col="#FFA5007D",type="h")

points(Area_Data49$Number,Area_Data49$Dev_3AgeStrat, col="#FFA5007D",type="h")

points(Area_Data67$Number,Area_Data67$Dev_3AgeStrat, col="#FFA5007D",type="h")

legend("topright",lty=1,col=c("black","#FFA5007D"),legend=c("Ad Hoc","Age-balanced"),title="Model",cex=1.1,bty="n")
